# Patient reported outcomes in head and neck cancer: selecting instruments for quality of life integration in clinical protocols

**DOI:** 10.1186/1758-3284-2-32

**Published:** 2010-10-31

**Authors:** Augusta P Silveira, Joaquim Gonçalves, Teresa Sequeira, Cláudia Ribeiro, Carlos Lopes, Eurico Monteiro, Francisco L Pimentel

**Affiliations:** 1 Oral Anatomy and Oral Histology- Health Sciences Department, Fernando Pessoa University, Rua Carlos da Maia, 296, 4200-150 Porto, Portugal; 2 Institute for Biomedical Sciences Abel Salazar- Porto University, Lg. Prof. Abel Salazar no. 2. 4099-003 Porto, Portugal; 3 The Centre of Health Studies and Research of the Coimbra University, Av. Dias da Silva, 165, 3004-512, Coimbra, Portugal; 4 Math Department, Polytechnic Institute of Cávado and Ave, Campus do IPCA - Lugar do Aldão 4750-810 Vila Frescainha S. Martinho Barcelos, Portugal; 5 Institute for Molecular and Cell Biology, Rua do Campo Alegre, 823, 4150-180, Porto, Portugal; 6 Health Sciences Department, Portuguese Catholic University, Campus Viseu Estrada da Circunvalação; 3504-505, Viseu, Portugal; 7 Santiago de Compostela University - Facultad De Medicina Y Odontologia Rua San Francisco, S/N, 15704, Santiago De Compostela, Espanha; 8 Health Sciences Department, Institute for Biomedical Sciences Abel Salazar- Porto University, Lg. Prof. Abel Salazar no. 2. 4099-003 Porto, Portugal; 9 Fernando Pessoa University, Rua Carlos da Maia, 296, 4200-150 Porto, Portugal; 10 Portuguese Institute for Oncology - Porto, Otorhinolaringology service (IPO-Porto, ORL). Rua Dr. António Bernardino de Almeida, 4200-072, Porto, Portugal; 11 Aveiro University, Secção Autónoma Ciências da Saúde; Campus Universitário de Santiago, Aveiro, Portugal Infante D. Pedro Hospital, Aveiro, Av. Artur Ravara, 3814-501, Aveiro, Portugal

## Abstract

**Background:**

Health Related Quality of Life has been used in medical research for more than twenty years, being progressively accepted during the last decade as an important patient reported outcome. Considering the multidimensional approach involved in Health Related Quality of Life assessment, instrument applicability and cultural adaptation must be tested for each population. In order to select the most appropriate instrument for Head and Neck cancer patients, two major Health Related Quality of Life specific questionnaires for Head and Neck cancer patients were compared. Conceptual differences, psychometric characteristics, scores, reliability, construct validity and sensitivity to symptomatology, tumour location, tumour size were analyzed.

**Methods:**

102 consecutive Head and Neck cancer patients completed two different Health Related Quality of Life questionnaires: EORTC QLQ-C30 and its specific head and neck module QLQ-H&N35 and the Functional Assessment of Cancer Therapy Scales (FACT-H&N). Patients completed the questionnaires, immediately before consultation as a part of the routine evaluation.

**Results:**

A greater variability was always found in the EORTC QLC-C30 questionnaire's scores for all comparable domains. Both instruments revealed a good internal consistency and demonstrated to be good tools to distinguish symptomatic patients. The EORTC questionnaires still demonstrated sensitivity to distinguish T3 and T4 staging. Conceptual differences and the psychometric characteristics are discussed. Our results suggest that these two instruments assess different aspects of Health Related Quality of Life - the questionnaires should be used separately and chosen according to the study objectives and methodology.

**Conclusions:**

This study emphases the importance in selecting the appropriate tool as a critical success factor in implementing routine Health Related Quality of Life assessment in clinical practice. This decision assumes particularly importance when utilization of results in real time and integration into clinical protocols are considered.

## Background

Health related Quality of Life Quality of life (HRQoL) is by definition a multi-dimensional global construct, introduced as keyword in the United States National Library of Medicine in 1977 [1,2]. HRQoL, has been used in medical research for more than twenty years, and has been introduced in clinical practice as an important outcome parameter in present medicine practice according to the contemporary holistic approach to the patient [3,4].

Over the past 10 years, HRQoL has been progressively more accepted as an important patient outcome result in oncology along with the other conventional outcomes used before such as treatment success, mean survival, disease free survival or cancer controlled survival [5,6].

### Importance of Health Related-Quality of Life assessment in head and neck cancer patients

Head and neck cancer is undoubtedly related to a decrease in HRQoL. After diagnosis, the treatment most frequently determines a deterioration of basic functions such as breathing, mastication, salivating, swallowing and speaking. Sense's impairment such as hearing, taste and smell along with possible esthetics changes will promote a negative impact in both patients and their relatives HRQoL [1,4,7].

HRQoL assessment allows head and neck cancer patients careful monitoring, may recognize risk patient groups being predictive for time to progression suggesting this evaluation as a new prognostic marker for survival [8-10]. HRQoL questionnaires enable patient's clustering according to their most frequent health concerns, ranking its intensity [1,11]. Also, HRQoL assessment can be considered a stimulating approach for effectiveness and the cost-effectiveness promotion (survival and quality-adjusted survival) contributing thus to the construction of an economical decision model [12-14].

### Instruments for HRQoL assessment in head and neck cancer patients

HRQoL instruments must exhibit comproved psychometric characteristics, such as: consistency, reliability, reproducibility, validity and sensibility to change.

Considering the multidimensional approach involved in the HRQoL assessment, instrument applicability and cultural adaptation must be tested for each population.

The instruments that fulfill the minimal requisites for Oncology HRQoL assessment include: Breast Cancer Chemotherapy Questionnaire (BCQ), Cancer Rehabilitation Evaluation Systems (CARES), European Organization for Research and Treatment of Cancer Quality of Life Questionnaire (EORTC-QLQ), Functional Assessment of Cancer Therapy Scales (FACT), Functional Living Index Scale (FLIC), Linear Analogue Self-Assessment, Medical Outcome Study Short Form (MOS SF-36), Multidimensional Quality of Life Scale, Quality of Life Index (QL- Index) (Spitzer Index), Rotterdam Symptom Check List (RSCL) [5,15,16]. The questionnaires EORTC-QLQ C30, FACT-G, MOS SF-36 and FLIC stand out as the main general in oncology.

There are specific instruments for head and neck cancer patients HRQoL evaluation such as: the EORTC QLQ-H&N35, the FACT H&N, the FLIC, the University of Washington Quality of Life Questionnaire (UW-QOL), the University of Michigan Head and Neck Quality of Life Questionnaire (HNQOL), the Head and Neck Radiotherapy Questionnaire (HNRQ), the Performance Status Scale-Head and Neck-(PSS-H&N), the Obturator Functioning Scale (OFS), the Late Side Effects on Daily Life Scale and the Oral and Pharyngeal Nursing Care Questionnaire (OPNCQ) [17-23].

### Clinical trials and clinical practice

HRQoL has been used as a health outcome measure mainly associated to clinical trials. The implementation of routine HRQoL assessment in clinical practice can be used for scientific documentation as well for clinical settings [24-26]. This advance is fundamental to obtain clinical meaningful data that can be a helpful outcome considering patients undergoing cancer treatments and, particularly, when additional supportive services and symptom management are concerned. Moreover, the careful HRQoL monitoring of cancer patients may identify potentially unmet needs and generate the basis of a stepped care model [26-28]. HRQoL information can thus support clinical decisions and promote health gains. However, results obtained either from clinical practice or research must be interpreted, not only in statistical terms, but also considering the clinical importance - it is required then an wide comprehension of the relationship between the patients outcome results and the patients perception of the change [29,30].

The present study aims to compare the two major HRQoL questionnaires specific for head and neck cancer patients: the EORTC QLQ-C30 and its specific head and neck module QLQ-H&N35 and the Functional Assessment of Cancer Therapy Scales (FACT H&N) in the Portuguese Institute for Oncology, Porto (IPO-Porto). Conceptual differences, psychometric characteristics and scores are analyzed and discussed. A major attention is made on significant differences that can be related to clinical important data.

## Methods

### Patients

From September 2008 to January 2010, 102 outpatients admitted to the Otorhinolaringology service (ORL service) IPO-Porto, Portugal, completed two different HRQoL questionnaires, immediately before consultation as a part of the routine evaluation. The completion order was randomized. Inclusion criteria were a Karnofsky index above 30, age below 90 years, ability to understand written and spoken Portuguese and provision of written consent.

### Questionnaires

The questionnaires under comparison were from the European Organisation for Research and Treatment of Cancer (EORTC) and the Functional Assessment of Cancer Therapy (FACT), both targeted to cancer patients. HRQoL was assessed by general questionnaires, EORTC QLQ-C30 and Functional Assessment of Cancer Therapy - General (FACT-G), and its disease-specific modules for head and neck (FACT H&N) cancer patients was also considered, the QLQ-H&N35 and the FACT H&N, respectively. Scores and conceptual characteristics were compared: between the two core questionnaires - EORTC-QLQC30/FACT-G - and between their disease-specific modules/extensions for H&N patients - QLQ-H&N35/FACT H&N (FACT-G + 12 H&N-specific questions), respectively.

### EORTC QLQ-C30 and the disease-specific module QLQ-H&N35

The EORTC QLQ-C30 (version 3.0) is a questionnaire developed to assess the HRQoL of cancer patients. It consists of 30 questions: twenty four form nine multi-item scales presenting various aspects of HRQoL: five functional scales (PF, Physical functioning; SF, Social functioning; EF, Emotional functioning; RF, Role functioning; CF, Cognitive functioning), three symptom scales (fatigue, pain, nausea and vomiting) and a global condition (health and quality of life). The remaining six are single-item scales describing different cancer relevant symptoms. During the scoring procedure, raw EORTC QLQ-C30 scores are linearly transformed into 0 e100 scales. For global health status and the five functioning scales, a score of 100 corresponds to a high HRQoL. For financial difficulties and the eight symptoms, a score of 100 implies maximum difficulty or symptom burden. The additional module - QLQ-H&N35 (version 3.0) - is disease-specific for head and neck patients. It consists of 35 questions organized in seven symptoms multi-item scales (twenty four questions are presented) and eleven are single-item scales describing different specific concerns of these head and neck cancer patients.

### FACT-G and the disease-specific FACT H&N

The FACT H&N (version 4) is a multidimensional, self-report HRQoL instrument specifically designed for use with head and neck cancer patients. It consists of 27 core items - FACT-G - assessing patient function in four domains: Physical, Social/Family, Emotional, and Functional well-being (Pwb, Swb, Ewb, and Fwb, respectively). It is further supplemented by 12 site specific items for head and neck related symptoms assessment - FACT - (H&N-G). Each item is rated on a 0 to 4 Likert type scale, and then combined to produce subscale scores for each domain, as well as a global HRQoL score. Higher scores represent better QoL.

### Questionnaire completion

The autonomy expressed for questionnaire completion as well the time patient needed to complete both questionnaires - EORTC QLQ-C30 (version 3)/QLQ H&N35 and FACT H&N - was evaluated.

### Sociodemographic and clinical data

Clinical data - such as tumour location, performance status (Karnofsky index), tobacco habits and present symptomatology - as well socio-demographic data - age, gender and schooling years - were collected from the patient's clinical process and complemented, when needed, in semi structured interviews.

### Ethics

All patients gave their informed consent. The data were collected for research purposes as part of the routine evaluation. The Committee for Ethics in Medical Research approved the use of these data for research.

### Analysis Strategies and Statistics

Completed questionnaires were scored according to the developers' instructions.

For tool comparison, 4 similar domains were always considered in EORTC QLQ-C30 and FACT-G, respectively: PF/Pwb, EF/Ewb, SF/Swb and RF/Fwb.

Descriptive data are presented with means, SDs, medians, ranges, and proportions as appropriate.

### Instrument's scores characteristics

In probability theory and statistics, skewness is a measure of the asymmetry of the probability distribution of a real-valued random variable. Qualitatively, a negative skew indicates that the tail on the left side of probability density function is longer than the right side and the bulk of the values (including the median) lie to the right of the mean. A positive skew indicates that the tail on the right side is longer than the left side and the bulk of the values lie to the left of the mean. Kurtosis is a measure of the "peakedness" of the probability distribution of a real-valued random variable. Higher kurtosis means more of the variance is the result of infrequent extreme deviations, as opposed to frequent modestly sized deviations.

### Reliability

Reliability expressed as internal consistency is a measure of how well the items in a multi-item scale interrelate. This is usually assessed by computing Cronbach's coefficient (Cronbach's alpha). The score reflects both the number of items and the degree of correlation between items.

### Sensitivity

Sensitivity measures how well the instrument identifies differences between groups. An instrument with a high sensitivity is able to detect a relatively small difference with a modest sample size. Sensitivity is measured by comparing the scores of different groups of patients. In this study the patient population was divided: i) in two groups according to the expressed symptomatology (symptomatic and asymptomatic) ii) into five broad groups according tumour location (vocal cord neoformation, laryngeal neoformation, pharynx neoformation, pharynxlaryngeal neoformation, and tongue neoplasia), iii) into four broad groups according tumour size (T1, T2 T3 and T4) and iv) into four broad groups according age (less than 45 year old, 46-55 years old, 56-65 years old and older than 65). The scores were compared using the Mann-Whitney or Kruskal-Wallis tests. Based on clinical experience, significant differences between these patient groups were expected.

### Construct Validity

Construct validation evaluates how well an instrument measures the construct it is intended to measure. Convergence between instruments (external convergent validity) is an assessment of the correlation between EORTC and FACT measures of the same concept. This is included in the multi-trait multi-method (MTMM) analysis where Pearson's correlations are used to compute the degree of correlation. A correlation above 0.70 between scales measuring the same concept is considered to be an indication of the same underlying concept. Discriminant validity was analysed to verify if the HRQoL operationalization does not correlate with other operationalizations that theoretically should not be correlated with.

## Results

### Patients Characteristics

One hundred two patients with median age of 59.4 years (range, 22-90 years) participated in this study by completing the questionnaires. The patient characteristics are summarized in Table 1.

**Table 1:** Sociodemographic and clinical characteristics of the participants (n = 102)

Categories		n (%)
		
**Gender**	Male	88 (86.3)
	Female	14 (13.7)
**Schooling (years)**	None	4 (3.9)
	1-4	70 (68.6)
	5-9	11 (10.8)
	10+	17 (16.7)
**Tumour location**	Vocal cord	18 (18.8)
	Laryngeal	17 (17.7)
	Pharynx	13 (13.5)
	Pharynx Laryngeal	6 (6.3)
	Tongue	5 (5.2)
	Others	37 (38.5)
**Tumour diagnosis**	Spinocellular carcinoma	77 (75.4)
	Displasic premalignant lesions	3 (3)
	Other diagnosis	15 (14.7)
	No diagnosis	7 (6.9)
**Symptomatology**	Symptomatic	53 (52.0)
	Asymptomatic	32 (31.4)
	Missing	17 (16.7)

Patients presented 59.4 ± 12.1 years and revealed a low educational level presenting 5 ± 3 schooling years and long tobacco habits being the majority (60.6%) exposed for more than 31 years.

The Karnofsky performance status scale revealed that all patients performed above 50% and 57% of head and neck cancer patients were mainly ascribed to high Karnofsky Index (90-100%).

### Questionnaire completion

The time response varied between 3 and 20 minutes (9,3 ± 3,1) for EORTC QLQ-C30 (version 3)/QLQ H&N35 and 3 to 23 minutes (7,3 ± 2,7) for FACT-H&N. Most head and neck cancer patients (79,4%) required some kind of help to answer the questionnaires.

### Questionnaire's conceptual characteristics

Main features of the two instruments used in this study are depicted in Table 2. It is observed EORTC questionnaires are always longer, both core (30 compared to 27 in FACT-G) and disease specific modules (35 and 12 in FACT- (H&N-G)). EORTC is always composed of scales and simple item questions but the disease-specific module in FACT is composed of simple items.

**Table 2:** Relevant instrument's characteristics.

	EORTC-QLQ C30	FACT-G	EORTC H&N35	FACT- (H&N-G)
**No. of items**	30	27	35	12
**Global score**	No	Yes	No	Yes
**Scales**	5 functional	4 functional	7 symptom	None
	3 symptom			
	2 global			
**Simple items**	6	0	11	12

EORTC QLQ-C30, European Organisation for Research and Treatment of Cancer Quality of Life - Core 30.

EORTC H&N35, European Organisation for Research and Treatment of Cancer, Head and Neck

FACT-G, Functional Assessment of Cancer Therapy - General.

FACT H&N, Functional Assessment of Cancer Therapy, Head and Neck

### Instrument's scores characteristics

A greater variability was always found in the EORTC QLC-C30 questionnaire's scores for all comparable domains - PF and Pwb, EF and Ewb, SF and Swb and finnally RF and Fwb. Asymmetry and flatness measures found in PF and Pwb evidenced great similarity within tools with the distribution tending to the left (-0.78 and -0.77, respectively) and a relatively flat curve indicating a platokurtic distribution (-0.22 versus -0.27) as illustrated in Table 3.

**Table 3:** EORTC QLQ-C30 and FACT -G characteristics: mean scores, standard deviation, skweness and kurtosis measurements and Cronbach's Alpha

Instrument	Scale	Mean	(SD)	Skewness	Kurtosis	Cronbach's Alpha
**EORTC QLQ-C30**	**Physical functioning**	0.69	0.27	-0.78	-0.27	0.87
	**Emotional functioning**	0.63	0.24	0.14	0.39	0.46
	**Social functioning**	0.68	0.25	-0.26	-0.84	0.89
	**Role functioning**	0,55	0.33	-0.48	-0.85	0.96
	**Cognitive functioning**	0.80	0.20	-1.78	1.13	0.27
	**Global Health Status**	0.61	0.23	-0.36	-0.38	0.92
	**Fatigue**	0.36	0.29	0.70	-0.43	0.85
	**Nausea/vomiting**	0.10	0.21	2.50	5.90	0.79
	**Pain**	0.33	0.28	0.68	-0.09	0.85
	**Dyspnoea**	0.19	0.28	1.34	0.85	-
	**Insomnia**	0.36	0.33	0.52	-0.79	-
	**Appetite loss**	0.22	0.33	1.20	0.25	-
	**Constipation**	0.15	0.26	1.73	2.37	-
	**Diarrhea**	0.03	0.10	2.15	7.73	-
	**Financial difficulties**	0.31	0.28	0.47	-0.70	-
**FACT-G**	**Physical well-being**	0.71	0.22	-0.77	-0.22	0.89
	**Social/family well-being**	0.66	0.17	-0.67	-1.23	0.71
	**Emotional well-being**	0.62	0.17	0.09	-0.57	0.69
	**Functional well-being**	0.51	0.19	0.18	0.73	0.84
	**Total**	0.63	0.15	-0.24	-0.50	0.76

Concerning SF and Swb, the most evident difference was the extent of the flattening where FACT-G showed a leptokurtic distribution (-0.67) and EORTC QLQ- C30 a platikurtic one (-0.84). The EF and Ewb domain presented similar measurement's distribution and may be considered as normal; RF and Fwb revealed a similar distribution in shape. The Cognitive domain exhibited a distribution very skewed to the right (-1.78).

When the disease-specific module EORTC QLQ- H&N35 scales are considered, it was found a wide score distribution as illustrated in Table 4. Less sexuality and Senses Problems scores were found to be right skewed (1.60 and 1.42, respectively).

**Table 4:** EORTC H&N35 and FACT - (H&N-G) characteristics: mean scores, standard deviation, skweness and kurtosis measurement, and Cronbach's Alpha.

Instrument	Scale	Mean	(SD)	Skewness	Kurtosis	Cronbach's Alpha
**EORTC H&N35**	**Pain**	0.25	0.22	0.92	0.32	0.72
	**Swallowing**	0.28	0.29	0.83	-0.34	0.90
	**Senses problems**	0.20	0.30	1.42	0.85	0.70
	**Speech problems**	0.35	0.34	0.79	-0.20	0.46
	**Trouble with social eating**	0.24	0.29	1.14	0.25	0.92
	**Trouble with social contact**	0.20	0.22	1.31	1.17	0.86
	**Less Sexuality**	0.16	0.26	1.60	1.73	0.99
	**Total**	-	-	-	-	0.90
**FACT (H&N-G)**	**Total**	-	-	-	-	0.57

### Reliability

When internal consistency was compared by measuring how well the items interrelate, EORTC demonstrated a higher total consistency, both in the core questionnaire EORTC QLQ-C30 (α = 0.87 versus 0.76 for FACT-G) as well in the disease specific module H&N35 (α = 0.90 versus 0.79 for FACT - (H&N-G)). Most values observed (Table 3, 4) were above 0.7 although the EF scale was found below in both questionnaires, EORTC QLQ-C30 and FACT-G (α = 0.46 and 0.69, respectively). Cognitive functioning scale revealed a low reliability (0.27).

The disease-specific module H&N35 revealed an internal consistency in all scales (α = 0.72 to 0.99) except in Speech problems (α = 0.46).

### Patient symptoms Sensitivity

Both core instruments detected significant differences between symptomatic and asymptomatic patients. EORTC QLQ-C30 detected differences in all scores except the EF scale and the single items Insomnia and Diarrhea. The symptomatic scores were always lower being Fatigue and RF the exceptions. FACT-G revealed to be sensitive in all domains except the Swb (Table 5).

**Table 5:** Scores of the four corresponding HRQoL scales of EORTC QLQ-C30 and FACT-G for the two group of patients: symptomatic and asymptomatic

EORTC and FACTG scores	Symptomatic	Asymptomatic	Mann Whithney (*p value*)
Mean (SD)	62% (n = 53)	38% (n = 32)	
**EORTC PF**	35.64	55.19	0.000
**FACT-G Pwb**	33.77	58.28	0.000
**EORTC SF**	36.32	53.56	0.001
**FACT-G Swb**	40.50	47.14	0.225
**EORTC EF**	39.75	48.39	0.115
**FACT-G Ewb**	58.33	33.75	0.000
**EORTC RF**	51.78	37.70	0.008
**FACT-G Fwb**	57.44	34.28	0.000

PF = Physical function, SF = Social function, EF = Emotional function, R = Role function

Pwb = Physical well-being, Swb = Social well-being, Ewb = Emotional well-being, Fwb = Functional well-being

Disease specific module EORTC QLQ-H&N35 was also able to discriminate in all scales except Senses problems nor three simple item questions considering Dry mouth, Weight loss and Weight loss. FACT - (H&N-G) was found to be sensitive when total score was considered and statistical significant differences were found in 5 single items (questions 1, 5, 7, 10 and 11).

### Tumour location sensitivity

EORTC QLQ-C30 revealed no sensitivity to tumour location being Fatigue in the discrimination boundary for Vocal cord neoformation (p = 0.05). The Pwb in FACT-G is sensitive to Vocal cord neoformation location (p = 0.008) presenting higer HRQoL score.

Vocal cord neoformation location was also positively discriminated when Swallowing, Trouble with social eating, and in the simple item Opening mouth were compared in EORTC QLQ- H&N35. FACT - (H&N-G) was found to sensitive when total score was considered and identified the pharynx neoformation location by the low scores found in the single items 1, 2, 5, 6 and 11. (Table 6)

**Table 6:** Scores of the corresponding HRQoL scales of EORTC QLQ-C30 and FACT-G for the five group of patients according to their tumour location

EORTC and FACTG scores	Vocal cord	Laryng	Pharynx	Pharynx laryngeal	Tongue	Kruskal-Wallis (*p value*)
Mean (SD)	18.8% (n = 18)	17,7% (n = 17)	13.5% (n = 13)	6,3% (n = 6)	5.2% (n = 5)	
**EORTC PF**	35.72	26.24	26.38	29.92	31.70	0.481
**FACT-G Pwb**	40.44	29.62	17.65	30.08	25.7	0.008
**EORTC SF**	34.28	27.91	24.23	30.50	36.10	0.438
**FACT-G Swb**	27.81	34.03	34.62	27.92	14.70	0.172
**EORTC EF**	31.14	29.50	28.27	31.42	30.40	0.992
**FACT-G Ewb**	34.11	34.09	23.25	24.17	25.60	0.286
**EORTC RF**	35.11	28.26	25.96	28.00	30.40	0.601
**FACTG Fwb**	34.36	33.44	22.08	21.17	33.80	0.166

PF = Physical function, SF = Social function, EF = Emotional function, R = Role function

Pwb = Physical well-being, Swb = Social well-being, Ewb = Emotional well-being, Fwb = Functional well-being

### Tumour size sensitivity

Patients with different tumour size are not depicted by the FACT-G assessment and EORTC QLC-C30 is only able to discriminate T3 and T4 when SF or the 2 simple items Diarrhea and Financial difficulties are considered.

The EORTC QLQ- H&N35 scales Swallowing and Trouble with social contact were found to be sensitive for T3 and T4, the same tumour size identified by FACT - (H&N-G) when total score and the single item questions 5 and 11 are addressed. Patients with T3 and T4 always presented the lowest scores. (Table 7)

**Table 7:** Scores of the corresponding HRQoL scales of EORTC QLQ-C30 and FACT-G for the four group of patients according to their tumour size

EORTC and FACTG scores	T1	T2	T3	T4	Kruskal-Wallis (*p value*)
Mean (SD)	10,8 % (n = 11)	12,7 % (n = 13)	17,6 % (n = 18)	31,4 % (n = 32)	
**EORTC PF**	39.55	35.12	43.19	34.56	0.544
**FACT-G Pwb**	45.95	36.19	38.53	34.55	0.493
**EORTC SF**	47.27	47.15	29.92	34.48	0.037
**FACT-G Swb**	42.18	30.23	36.75	39.27	0.515
**EORTC EF**	42.55	42.08	37.75	33.77	0.533
**FACT-G Ewb**	41.82	38.15	38.08	35.42	0.855
**EORTC RF**	38.00	40.96	39.81	34.63	0.753
**FACTG Fwb**	50.55	41.27	37.64	31.41	0.070

PF = Physical function, SF = Social function, EF = Emotional function, R = Role function

Pwb = Physical well-being, Swb = Social well-being, Ewb = Emotional well-being, Fwb = Functional well-being

### Construct Validity

#### Internal convergent validity

The EORTC QLQ C-30's revealed two correlations, between scales PF - RF (0.79) and RF - SF (0.72), whereas only one was found in FACT-G - between Fwb and Pwb scales (0.70).

#### External convergent validity

The four domains covered by both HRQoL instruments are illustrated in the MTMM correlation matrix shown in Table 8.

**Table 8:** MTMM Correlation Matrix

	PF	Pwb	SF	Swb	EF	Ewb	RF
**Pwb**	0.80						
**SF**	0.62	0.70					
**Swb**	0.19	0.19	0.21				
**EF**	0.42	0.49	0.48	0.18			
**Ewb**	0.51	0.67	0.54	0.35	0.63		
**RF**	0.79	0.75	0.72	0.18	0.44	0.56	
**Fwb**	0.71	0.70	0.65	0.39	0.43	0.60	0.70

EORTC QLQ-C30: PF = Physical function, SF = Social function, EF = Emotional function, RF = Role function; FACT-G: Pwb = Physical well-being, Swb = Social well-being, Ewb = Emotional well-being, Fwb = Functional well-being;

A convergence between instruments was found for PF and Pwb scales (0.80), PF and Fwb scales (0.71), SF and Pwb scales (0.70), PF and Fwb scales (0.71).

Both Physical and Role functions depicted in EORTC assessment revealed to correlate with either FACT-G Physical and Functional well-being (0.80 for Pwb, 0.71 for Fwb and 0.75 for Pwb, 0.70 for Fwb, respectively). Thus, for these two functional dimensions the two questionnaires seem to assess in a similar way. In opposition the Social and Emotional scales were found to evaluate the same issues but in a different way - Social function revealed a correlation with the Physical well-being (0.70) and no correlation were found for the Emotion function.

## Discussion

Head and neck cancer affects mainly men (ratio male : female ranges between 2 - 5:1, depending on tumour location), contributing to 4% of deaths among males in Portugal and represents the fifth cause of death with cancer in men [31,32]. In the present study it was found that 86.3% of cancer patients were men, long time smokers with low literacy. The most frequent tumour locations identified is in agreement with literature identifying the larynx as the most common site for head and neck cancer, being squamous cell carcinoma the preferential histopathology diagnostic[33-35].

Most cancer patients (54%) were in a symptomatic phase although a high Karnofsky Index (90-100%) was reported. These data emphases that the viewer's perception is different from the patient self-perception obtained by HRQoL assessment [36,37].

The instrument's characteristics analysis revealed that syntax is different between questionnaires: EORTC proposes a mixed order of questions and FACT-G chooses preferably statements organized into modules. The content of the EORTC questionnaires focuses on the everyday situations observing mainly physical and symptoms, and the FACT-G explores the existential problems and personal satisfaction. The EORTC version used head not a total score as FACT-G but presented a richer specific questionnaire for head and neck cancer patients.

Although the EORTC questionnaires- QLQ C30 and H&N 35- were administered separately and FACT H&N just once (it includes the core questionnaire plus the H&N specific module), more unanswered items (missing data) were obtained with FACT-H&N being the sexuality items the lesser answered. The fact that FACT-H&N considers these items answer optional, may encourage patients to not answer such questions and justify these results, which are consistent with other studies reporting a large proportion of missing data when considering the Sexuality item [38,39]. Asymmetry and flatness measures do not differentiate the questionnaires although EORTC QLQ-C30 flattening measure revealed to be closer to a normal distribution.

The higher variability found in EORTC QLQ- C30 functional scales and the leptokurtic distribution of Swb dimension in FACT-G suggest that when answering FACT-G questionnaire, patients tend to respond in the core values, reducing thus the effect of the measure.

Although both instruments showed good internal consistency it was found to be higher in the EORTC questionnaires. Low reliability in Cognitive Function scale from EORTC QLQ- C30 and FACT-(H&N-G) may be explained by the conceptual characteristics of these instruments. Both are good tools to distinguish symptomatic patients, however, both questionnaires show similar inefficiency differentiates tumour location. The EORTC questionnaires also revealed sensitivity to distinguish the two staging T3 and T4.

Just like other authors, it was found that administration of both questionnaires proved to be feasible, with acceptable response times. The easy, acceptable and understandable questionnaires format may facilitate its potential inclusion in routine clinical protocols [38-40].

## Conclusions

The data demonstrate that the EORTC QLQ-C30 (version 3) and EORTC QLQ-H& N35 and FACT H&N (Version 4) are good tools for HRQoL assessment in head and neck cancer patients, although the psychometric characteristics - fidelity, validity and sensitivity - are different.

Thus, our results suggest that these two instruments assess different aspects of QoL. The EORTC QLQ-C30 questionnaire provides a vision focused on physical and symptoms aspects, while the FACT H&N gives a multidimensional view of the concept, a broader perspective and comprehensive description of different areas. These results agree in general with other HRQoL instruments comparative studies and demonstrate that a tool does not replace the other and direct result comparison was not possible. The questionnaires should be used separately and chosen according to the study objectives and methodology.

This study emphases the importance in selecting the appropriate tool as a critical success factor in implementing routine Health Related Quality of Life assessment in clinical practice. This decision assumes particularly importance when utilization of results in real time and integration into clinical protocols is considered.

## Competing interests

The authors declare that they have no competing interests.

## Authors' contributions

AS conceived of the QoLIP, and Validation Model design, participated in the acquisition, analysis and interpretation of data, drafted and submitted the manuscript.

JG conceived of the QoLIP, and Validation Model design, participated in the acquisition, analysis and interpretation of data, and performed the statistical analysis. TS participated in the analysis and interpretation of data, helped to draft the manuscript and revised it critically for important intellectual content.

CR participated in the analysis and interpretation of data, and revised the manuscript. CL, EM and FLP conceived of the study, participated in its design and coordination, helped to draft the manuscript, and revised it critically.

All authors read and approved the final manuscript.
